# Influence of the use of autogenous bone particles to close the access window after maxillary sinus floor augmentation: an experimental study in rabbits

**DOI:** 10.1186/s40729-020-0206-2

**Published:** 2020-03-04

**Authors:** Giacomo Favero, Jose Viña-Almunia, Carmen Carda, José Javier Martín de Llano, Berta García-Mira, David Soto-Peñaloza, Miguel Peñarrocha-Diago, Daniele Botticelli

**Affiliations:** 1 Private practice, London, UK; 2 Oral Surgery Unit. Department of Stomatology, Faculty of Medicine and Dentistry, Clinica Odontológica, University of Valencia, Valencia, Spain; 3 Department of Pathology and Health Research Institute of the Hospital Clínico (INCLIVA), Faculty of Medicine and Dentistry, University of Valencia, Valencia, Spain; 4 Ciber-BBN, Instituto de Salud Carlos III, Valencia, Spain; 5 ARDEC Academy, Rimini, Italy

**Keywords:** Sinus floor elevation, Xenograft, Autogenous bone, Collagen membrane, Osteoconductivity

## Abstract

**Aim:**

To study the influence on the healing of the placement of particulate autogenous bone in the antrostomy and in the subjacent region after maxillary sinus elevation.

**Material and methods:**

Sixteen New Zealand rabbits were undergone to bilateral maxillary sinus floor augmentation with 4 × 4 mm antrostomy dimension. The sinus mucosa was elevated, and the space obtained was filled with xenograft. In the test site (treated sites), autogenous bone was harvested from the tibia and was placed either in the antrostomy and the subjacent region while the control site was left untreated. Antrostomy was covered bilaterally with collagen membranes. Animals were euthanized after 1 and 8 weeks of healing, with 8 rabbits in each group. Histomorphometric evaluations were done. The Wilcoxon test is used for statistical analysis, for a 5% statistical significance.

**Results:**

After 1 week of healing, the new bone proportion in the antrostomy was 7.7 ± 11.2% and 6.1 ± 6.4% in the treated and untreated sites, respectively. In the subjacent region (close-to-window region), hardly any new bone was assessed. In the elevated region, 2.7–2.8% of total new bone was found in both sites. In the antrostomy region, after 8 weeks of healing, 35.5 ± 20.9% of new bone in the treated sites, and 28.6 ± 24.1% in the untreated sites was observed (*p* = 0.499). In the close-to-window region, the respective proportions were 25.8 ± 16.1% and 17.6 ± 16.3% (*p* = 0.018). In the elevated region, the total new bone reached fractions of 27.9 ± 12.9% and 23.6 ± 15.2% in the treated and untreated sites, respectively (*p* = 0.128).

**Conclusions:**

The placement of autogenous bone in the antrostomy and the subjacent region after maxillary sinus elevation, slightly enhanced bone formation compared with sites only grafted with xenograft. Though, only the subjacent close-to-window region showed a statistical significance at 8 weeks of healing. Despite the limitations of the present study, due to its preclinical nature, findings should be extrapolated to humans with caution.

## Introduction

Maxillary sinus floor elevation through lateral access was first proposed in 1977 [1], while the technique was published in 1984 [2]. Several modifications in the surgical approach and the biomaterials used have been introduced over time [3–5]. In a systematic review with meta-analysis, it was concluded that the best survival rate was observed when implants with rough surface and membrane to cover the lateral window were used [3]. However, another systematic review with meta-analysis did not find a difference in survival rate for a lateral window with or without the protection of a membrane [5]. Moreover, clinical studies reported higher proportions of new bone at grafted sinuses protected by a collagen membrane compared to unprotected sites [6, 7], while in a systematic review with meta-analysis, no differences in bone formation were disclosed [8]. The use of a collagen membrane did not prevent the loss of biomaterial through the lateral window [9–11].

Nevertheless, the closure of the antrostomy with bone was documented by CBCTs taken after 9 months of healing [10, 11]. Even though the lateral window might be assessed as closed at the CBCTs analysis, a histological study in humans showed higher amounts of bone and bone marrow at biopsies taken from the grafted sites through the alveolar crest compared to those from the antrostomy region [12]. Moreover, some antrostomies presented incomplete healing of the region, with connective tissue interposed between the margin of the antrostomies that affected a complete closure and corticalization.

The repositioning of the bone window removed before sinus floor elevation from the antrostomy is another option that has been applied both in clinical [13, 14] and in experimental studies [15, 16], and optimal results have been reported. Though data have been reported on autogenous bone used alone or mixed with bone substitutes [14, 17]; no data have been reported on the use of autogenous bone only in the antrostomy and in the subjacent region.

Hence, the present experiment aimed to study the influence on healing, of the autogenous bone particle placement in the antrostomy and in the subjacent region after maxillary sinus elevation.

## Materials and methods

### Ethical statement

Prior to the experiment, the protocol was approved by the Ethics Committee of Valencia University, Spain (A1434714637496). The guidelines indicated by the Council Directive of the European Union (53/2013; February 1, 2013) for animal experimentation and the ethical rules proposed by Royal Decree 223, March 14 and October 13, 1988, were fulfilled. The study was reported following the ARRIVE guidelines.

### Study design and experimental animals

Sixteen New Zealand rabbits of about 24 weeks of mean age and 3 to 4 kg of weight were used for the experiment. The animals were divided into two groups, eight animals per group, based on the period of euthanasia that was fixed to 1 and 8 weeks from the surgery.

### Randomization and allocation concealment

The randomization was performed electronically (www.randomization.com) by an author that was not involved in the selection of the animals and in the surgical procedures (DB). The surgeon (JVA) was informed about the allocation treatment at the access window after having performed the sinus lift.

### Sample size

Data from a study in rabbits [18] showed differences in the bone formation of about 10%. Considering this value as clinically relevant, and supposing a slightly greater variability (standard deviation ± 6%), a power of 0.8, and α = 0.05, a sample of 7 animals was considered sufficient to disclose differences. The number was then increased to 8 to compensate for the possible loss of animals.

### Clinical procedures

Before surgery, a premedication was performed in all animals using 22 mg/kg of ketamine (Ketolar®; Pfizer, Madrid, Spain) and 2.5 mg/kg of xylazine (Rompun®, Bayer, Germany). As general anesthesia 1.5 mg/kg of propofol (Diprivan®, IL, USA) endovenous and maintained subsequently with isoflurane 2% and oxygen. During surgery, an intramuscular injection of 2.5 mg/kg of morphine 1%, (Braun®, Melsungen, Germany) was administered. As local anesthesia, 4% articaine with epinephrine 1/100.000 (ULTRACAIN DS forte®, Hoechst GmbH, Frankfurt, Germany) was used both in the tibia and in the nasal dorsum regions.

**Fig. 1:**
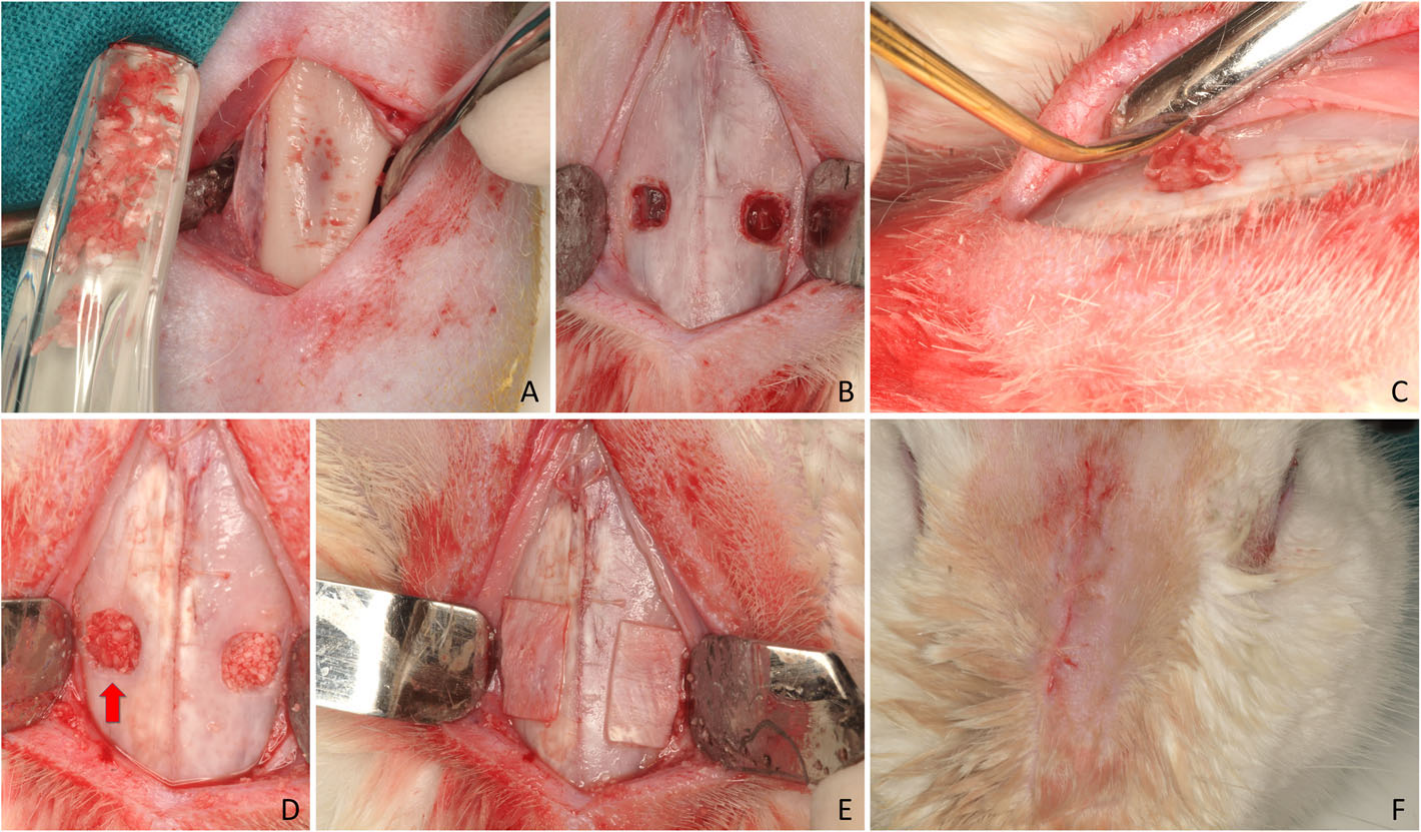
Clinical view of the surgical procedures. **a** Tibial bone exposed for autogenous bone harvesting using a bone scraper. **b** Antrostomies prepared. **c** Autogenous bone particles placed in the antrostomy. **d** Xenograft and bone particles (red arrow) at the antrostomies. **e** Collagen membranes placed on the antrostomies. **f** Wounds closed with sutures

The skin at the proximal tibia was shaved and disinfected with Betadine (MEDA Pharma S.L., Madrid, Spain), and an incision was performed several millimeters below the anterior tibial tuberosity. The flaps were elevated to expose the tibial bone of the proximal region (Fig. 1a). Bone particles were collected using a Savescraper Twist curve (CGM spa, Divisione Medicale META, Reggio Emilia, Italy) and maintained in saline. The wounds were sutured with nylon (Aragó®, Barcelona, Spain).

Afterward, a trichotomy was performed in the nasal dorsum and, after disinfection of the experimental region using Betadine (MEDA Pharma®, Madrid, Spain), a sagittal incision was carried out. The skin and the periosteum were dissected and shifted laterally to expose the nasal bone. Antrostomies, 4 × 4 mm in dimensions, located about 3–4 mm laterally to the midline and about 10 mm in front of the nasal-frontal suture, were prepared bilaterally grinding the bone with a round diamond bur (Fig. 1b), under conspicuous irrigation with saline.

The sinus mucosa was elevated from the bone walls with a small elevator (Bontempi®, San Giovanni in Marignano, RN, Italy), and the space obtained was grafted with a collagenated cortico-cancellous porcine bone (OsteoBiol Gen-Os, Tecnoss®, Giaveno, Italy; 250–1000 μm). The autogenous bone particles were placed in the antrostomy and the subjacent region at the randomly assigned sites (treated sites; Fig. 1c, d). Collagen membranes (OsteoBiol® Evolution 0.3 mm, Tecnoss®, Giaveno, Italy; Fig. 1e) were placed to cover the antrostomies both at the treated and untreated sites. Resorbable sutures (Vicryl®, Johnson-Johnson, New Brunswick, NJ, USA) were used to close both periosteum and skin (Fig. 1f).

### Maintenance care

Meloxicam (Normon®, Madrid, Spain) 0.2 mg/kg once a day for 7 days and Buprenorphine hydrochloride (Buprex®, Hull, UK) 0.02 mg/kg twice a day for 3 days were administered subcutaneously. The rabbits were kept in individual cages in controlled temperature rooms at the animal facilities of the University of Valencia. The monitoring of wounds and biological functions was performed for the whole follow-up.

### Euthanasia

The animals were euthanized using 50 mg/kg of sodium pentobarbital (Nembutal® Schaumburg, IL, USA).

### Paraffin sections preparation

The experimental region was dissected and fixed for 7 days at room temperature in formalin 10% and subsequently decalcified in Osteosoft (Merck KGaA®, Darmstadt, Germany). After decalcification, the specimens were washed in distilled water and included in paraffin. Sections of about 5 μm in width were prepared in a microtome (RM2245, Leica Biosystems, Wëtzlar, Germany). A section from the central region of the antrostomy was selected and stained with scarlet-acid fuchsine and toluidine blue and used for histomorphometric analysis.

### Histomorphometric analysis

Overlapping calibrated digital images of the tissues were recorded with Leica Applications Suite version 4.4.0 software from a bright field Leica DM4000 B microscope (Leica Microsystems GmbH, Wëtzlar, Germany) equipped with a 5× lens and DFC420 digital camera. Single images were pasted and merged to compose each elevated sinus using the program Photoshop (Adobe Photoshop CC 2015.0.0).

**Fig. 2:**
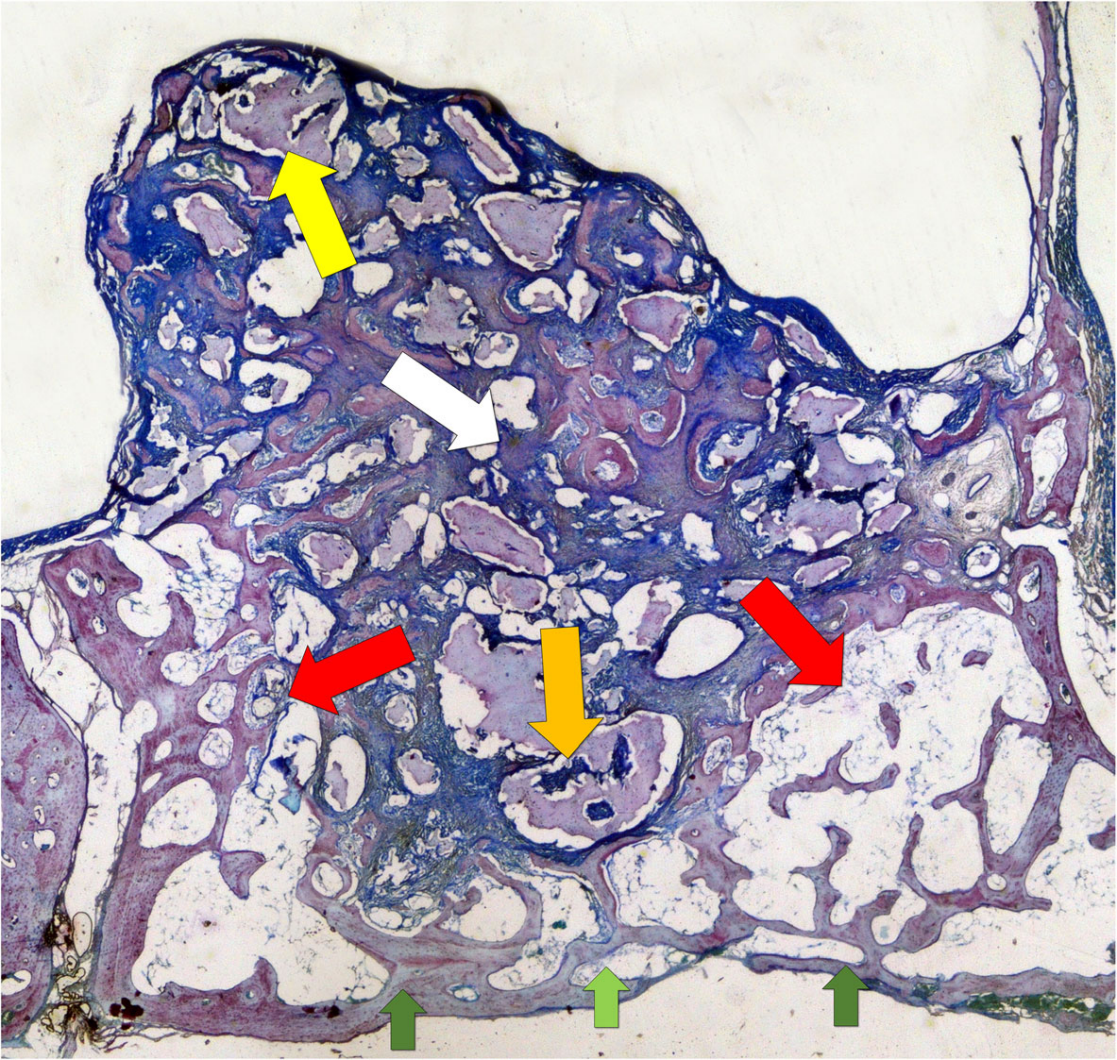
The various regions evaluated at the histomorphometric analyses. Bone walls (red arrow); middle (white arrow); sub-mucosa (yellow arrow); close-to-window (orange arrow). The antrostomy region was also evaluated at the medial and lateral edges (dark green arrows) and in the middle aspect (light green arrow)

The histomorphometric measurements were taken by an expert assessor (JM) using a lattice with squares of 75 μm. The following different areas were assessed within the grafted regions (Fig. 2): (i) close to the bone walls (Bone walls region), (ii) center of the augmented region (middle region), (iii) subjacent to the sinus mucosa (sub-mucosa region), (iv) and close to the antrostomy (close-to-window region). Moreover, the antrostomy (antrostomy region) was also evaluated in three different areas: adjacent to the medial and lateral edges, and the middle of the access window (Fig. 2). The tissues evaluated were newly formed bone, graft material, and soft tissues. Percentages were subsequently calculated.

### Data analysis

The sites that received autogenous bone in the antrostomy and the subjacent regions (close-to-window) were considered as a test (treated sites) while the sites that did not receive the autogenous bone were considered as control (untreated sites).

The primary variable was the new bone percentage in the region of major interest that was the antrostomy and close-to-window areas. The mean new bone within the augmented sinus was considered as a secondary variable. Mean values and standard deviations were calculated for each outcome variable. The results were calculated using the software Excel 2013 (Microsoft Corporation, Redmond, WA, USA), while the statistical analyses were performed with the IBM SPSS Statistics software v.19 (IBM Inc., Chicago, IL, USA). In the text are reported only mean values and standard deviations while the tables were complemented with 25th, 50th (median), and 75th percentiles were also added. The Wilcoxon test was used to evaluate differences between the two sites with a level of significance set at 5%.

## Results

Biopsies could be harvested from all animals. However, histological sections could not be obtained from one rabbit of the 8 weeks group; therefore, eight and seven were achieved for the 1-week and 8-week periods, respectively.

### Antrostomy and close-to-window regions

**Fig. 3:**
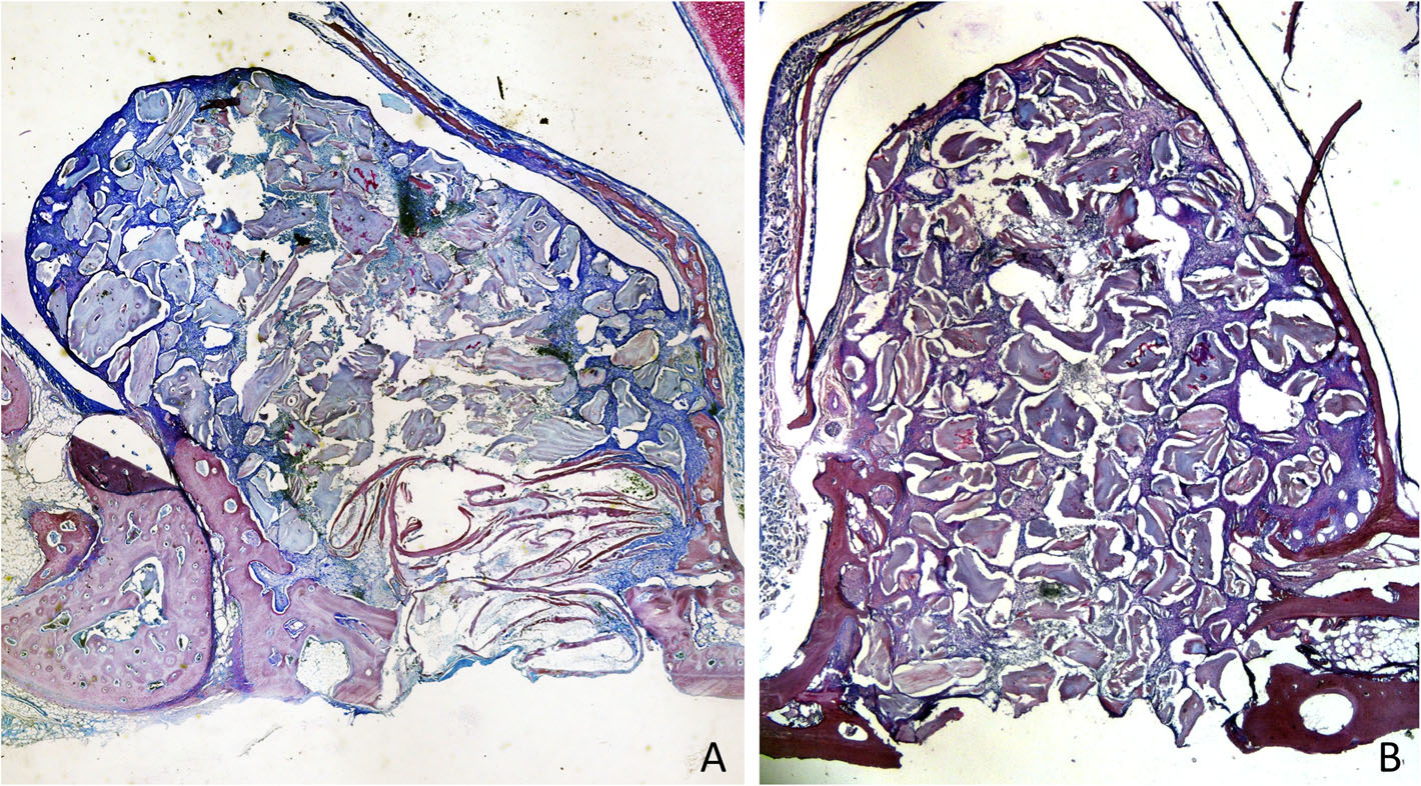
Photomicrographs of decalcified sections illustrating the healing after 1 week. **a** Treated site. Bone strips occupying the antrostomy and the subjacent area (close-to-window region). **b** Untreated site. Note the new bone-forming from the sinus bone walls. Scarlet-acid fuchsine and toluidine blue stain. Images grabbed at × 20 magnification

**Fig. 4:**
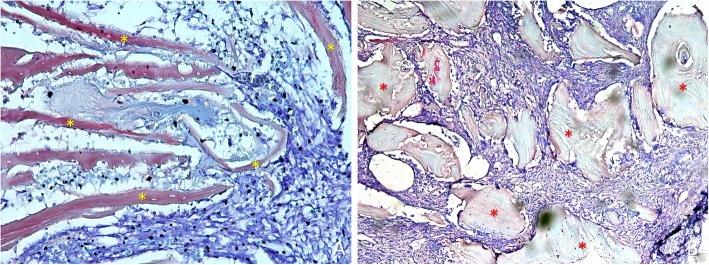
Photomicrographs of ground sections. **a**) Treated site. Bone residues (examples in yellow asterisks) included in soft tissue containing fibroblast-like cells and inflammatory cells. **b**) Untreated site. Xenograft residues (examples in red asterisks) surrounded by soft tissue rich in fibroblast-like cells. Scarlet-acid fuchsine and toluidine blue stain. **a**) 200 x magnification.; **b**) 100 x magnification

After 1 week of healing, at the treated sites, the antrostomy and close-to-windows regions were occupied by a high proportion of residues of autogenous bone (Fig. 3a), included in soft tissue containing fibroblast-like cells and inflammatory cells (Fig. 4a). At the untreated sites, a high amount of xenograft was found (Fig. 3b), surrounded by soft tissues rich in fibroblast-like cells, that appeared to densify around the particles (Fig. 4b). At the histological assessments, minimal amounts of bone were found after 1 week of healing, especially in close contact with the edges of the antrostomy. In the antrostomy (Table 1), the total amount of new bone was 7.7 ± 11.2% in the treated sites, and 6.1 ± 6.4% in the untreated sites (*p* = 0.889). In the close-to-window region, 0.6 ± 1% of new bone was found in the treated region, while no new bone was present in the untreated region (*p* = 0.109).

**Table 1:** Histomorphometric analysis. Tissues evaluated in the various regions after 1 week of healing

		Antrostomy			Sinus regions				
		Edges	Center	Total	Close-to-window	Middle	Sub-mucosa	Bone walls	Total
New bone	Treated sites	**9.2** ± 10.6 4.6 (3.2;12.5)	**5.2** ± 13.9 0.0 (0.0;0.5)	**7.7** ± 11.2 2.7 (1.9;7.9)	**0.6** ± 1.0 0.0 (0.0;0.7)	**0.0** ± 0.0 0.0 (0.0;0.0)	**1.4** ± 1.8 0.8 (0.0;2.2)	**7.7** ± 6.3 8.2 (1.3;11.1)	**2.8** ± 2.6 2.2 (0.7;4.0)
	Untreated sites	**8.9** ± 8.5 6.2 (4.0;10.8)	**1.0** ± 2.7 0.0 (0.0;0.0)	**6.1** ± 6.4 3.9 (2.6;6.9)	**0.0** ± 0.0 0.0 (0.0;0.0)	**0.0** ± 0.0 0.0 (0.0;0.0)	**1.0** ± 1.4 0.3 (0.0;1.3)	**8.1** ± 5.9 7.4 (3.4;11.3)	**2.7** ± 2.2 1.9 (1.0;3.2)
Autogenous	Treated sites	16.7* ± 15.0 15.0 (4.6;24.3)	25.6* ± 15.3 27.6 (17.1;33.9)	20.3* ± 14.1 23.4 (9.5;26.6)	18.4* ± 16.9 9.6 (7.3;27.3)	**0.2** ± 0.4 0.0 (0.0;0.1)	**0.0** ± 0.0 0.0 (0.0;0.0)	4.3* ± 5.1 1.9 (0.0;8.3)	4.2* ± 4.0 2.0 (1.5;6.7)
	Untreated sites	0.2* ± 0.6 0.0 (0.0;0.0)	0.0* ± 0.0 0.0 (0.0;0.0)	0.1* ± 0.4 0.0 (0.0;0.0)	0.1* ± 0.2 0.0 (0.0;0.0)	**0.0** ± 0.0 0.0 (0.0;0.0)	**0.0** ± 0.0 0.0 (0.0;0.0)	0.0* ± 0.0 0.0 (0.0;0.0)	0.0* ± 0.0 0.0 (0.0;0.0)
Xenograft	Treated sites	4.1* ± 6.4 1.1 (0.0;4.4)	4.0* ± 10.2 0.0 (0.0;1.3)	4.0* ± 7.5 0.9 (0.0;3.1)	21.3* ± 14.7 18.4 (14.5;23.2)	**52.8** ± 12.7 54.0 (39.9;61.3)	**53.9** ± 11.5 49.4 (45.1;63.5)	33.6* ± 8.5 35.0 (26.3;40.6)	42.3* ± 8.5 41.3 (36.3;47.0)
	Untreated sites	36.5* ± 15.6 44.5 (20.5;48.6)	55.3* ± 14.1 58.4 (43.6;63.8)	43.5* ± 14.1 45.6 (31.4;54.4)	55.9* ± 9.0 54.6 (51.6;58.9)	**59.5** ± 9.9 61.7 (50.2;67.5)	**56.8** ± 4.9 58.2 (55.8;59.7)	41.0* ± 9.2 44.0 (35.3;48.7)	52.5* ± 5.2 52.2 (50.5; 56.3)
Soft tissue	Treated sites	**70.0** ± 13.8 73.1 (65.7;77.0)	65.2* ± 15.4 62.1 (56.2;73.3)	**68.0** ± 13.5 68.9 (61.8;77.8)	**59.8** ± 16.0 63.3 (48.3;70.7)	**47.0** ± 12.5 45.9 (38.7;59.9)	**44.7** ± 11.8 49.9 (36.3;52.0)	**54.4** ± 9.6 54.4 (48.1;61.3)	50.7* ± 9.0 52.1 (46.7;58.4)
	Untreated sites	**54.3** ± 10.0 51.8 (47.8;59.0)	43.7* ± 13.2 41.6 (36.2;52.9)	**50.3** ± 9.6 50.8 (41.8;58.1)	**44.1** ± 8.9 45.4 (41.1;48.0)	**40.5** ± 9.9 38.3 (32.5;49.8)	**42.2** ± 4.3 41.5 (39.2;44.2)	**50.9** ± 7.1 *49.5* (46.5;53.9)	44.9* ± 5.0 46.5 (40.6;48.2)

Data in percentages. Mean values in bold ± standard deviations. Medians (25th; 75th) percentiles. *p < 0.05

**Fig. 5:**
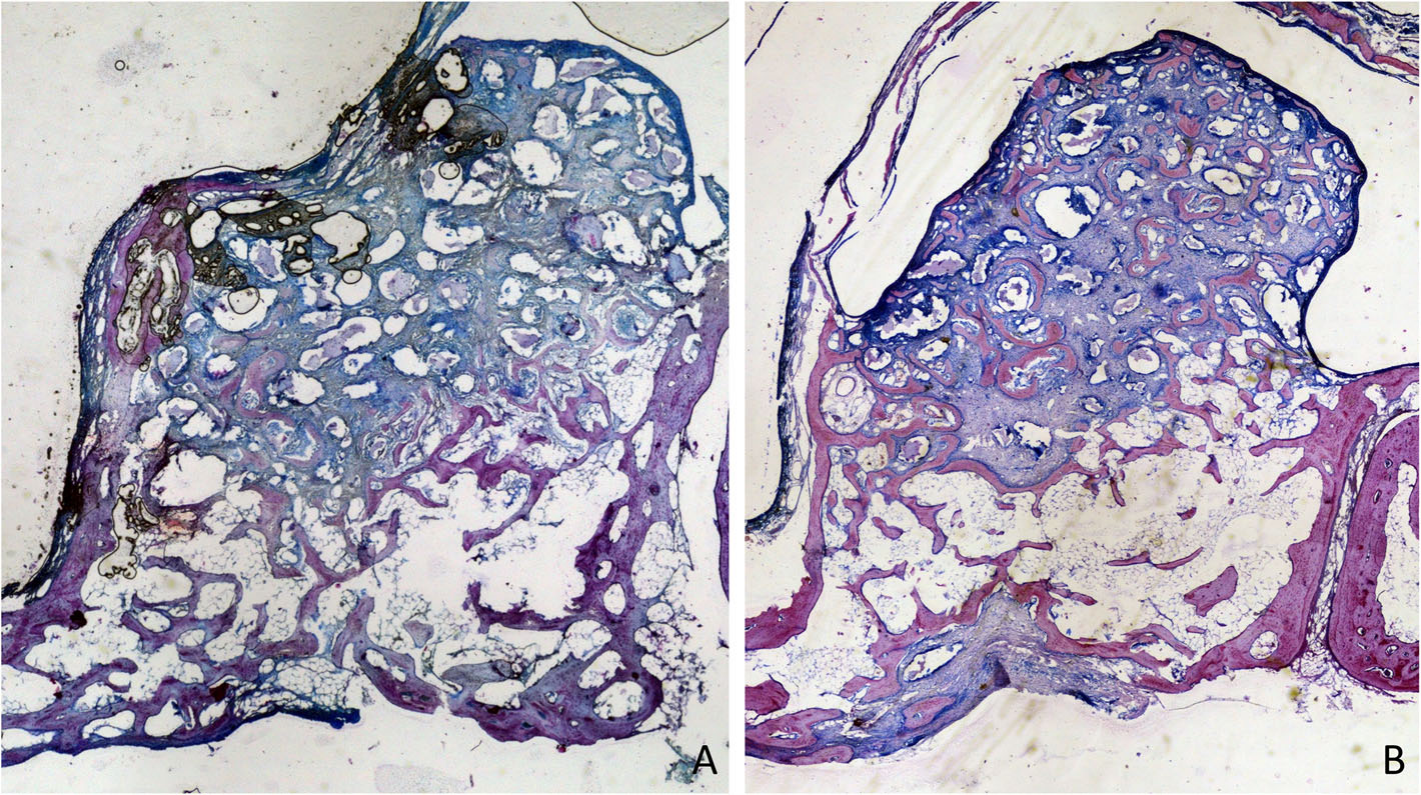
Photomicrographs of decalcified sections illustrating the healing after 8 weeks. Both at the treated (**a**) and untreated (**b**) sites, the antrostomy was closed in most cases, presenting residual defects of various dimensions in the outer side. New bone was connecting the lateral and medial sinus walls. The middle and sub-mucosa regions were not healed completely yet. Scarlet-acid fuchsine and toluidine blue stain. Images grabbed at × 20 magnification

**Fig. 6:**
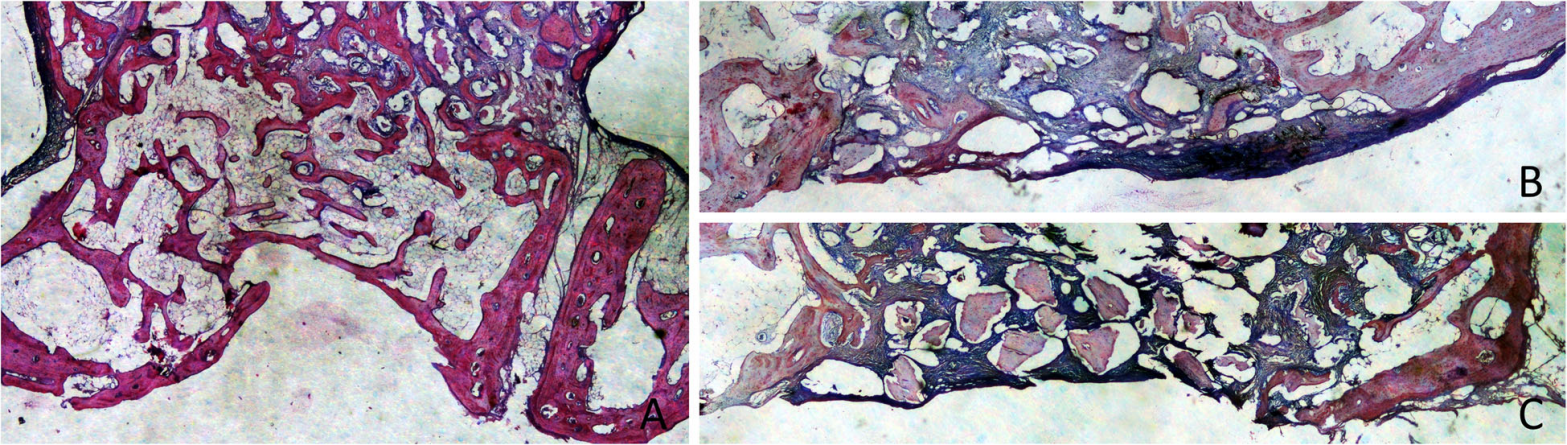
Photomicrographs of decalcified sections illustrating the healing after 8 weeks. **a** Treated site. Most of the antrostomies presented remaining defects in the outer contour. **b**, **c** Untreated sites. Two antrostomies of the treated sites and four of the untreated sites appeared not closed with corticalized bone and presented connective tissue interposed between the edges of the antrostomy. Scarlet-acid fuchsine and toluidine blue stain. **a** Image grabbed at × 20 magnification. **b**, **c** Images grabbed at × 40 magnification

**Fig. 7:**
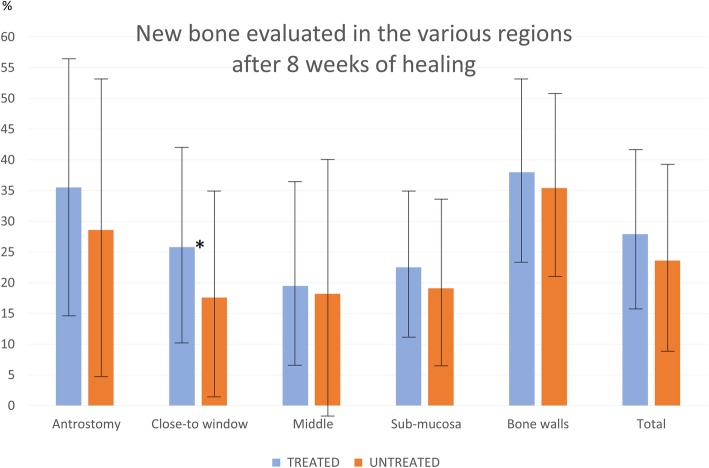
Box-plot representing the new bone percentage and standard deviations (whiskers) found in the various regions evaluated after 8 weeks of healing. (*), a statistical significant difference

After 8 weeks of healing, five antrostomies in the treated sites, and three in the untreated sites were repaired with corticalized bone (Fig. 5a, b). Most of the antrostomies presented the remaining defects in the outer contour located in the center (Fig. 6a). Two antrostomies of the treated sites and four of the untreated sites appeared not closed with corticalized bone and presented connective tissue interposed between the edges of the antrostomy (Fig. 6b, c). In the antrostomy region (Table 2; Fig. 7), the new bone was 35.5 ± 20.9% in the treated sites and 28.6 ± 24.1% in the untreated sites, being the difference not statistically significant (*p* = 0.499). In the close-to-window region, the new bone was 25.8 ± 16.1% and 17.6 ± 16.3% in the treated and untreated sites, respectively. The difference was statistically significant (*p* = 0.018).

**Table 2:** Histomorphometric analysis. Tissues evaluated in the various regions after 8 weeks of healing

		Antrostomy			Sinus regions				
		Edges	Center	Total	Close-to-window	Middle	Sub-mucosa	Bone walls	Total
New bone	Treated sites	*40.3* ± 21.3 37.8 (27.0;56.0)	*24.3* ± 23.2 22.0 (3.4;42.2)	*35.5* ± 20.9 27.7 (23.3;52.0)	25.8* ± 16.1 22.9 (15.2;39.7)	*19.5* ± 16.7 11.7 (10.1;22.3)	*22.5* ± 11.6 20.4 (12.6;31.3)	*38.0* ± 15.0 44.8 (31.8;47.5)	*27.9* ± 12.9 30.1 (19.6;34.5)
	Untreated sites	*32.2* ± 22.3 33.0 (12.5;44.9)	*20.5* ± 30.2 10.7 (0.0;24.6)	*28.6* ± 24.1 25.7 (8.8;39.4)	17.6* ± 16.3 21.8 (3.0;25.8)	*18.2* ± 21.4 5.7 (2.1;31.1)	*19.1* ± 13.5 13.1 (12.3;27.8)	*35.4* ± 15.0 38.3 (24.0;44.4)	*23.6* ± 15.2 22.6 (11.8;34.2)
Xenograft	Treated sites	*3.3* ± 5.8 0.0 (0.0;3.9)	3.6* ± 5.0 0.0 (0.0;6.7)	*3.4* ± 4.9 0.0 (0.0;5.6)	*15.5* ± 14.4 15.4 (2.1;27.5)	*18.8* ± 11.3 20.8 (11.8;26.1)	*21.1* ± 9.9 20.2 (18.3;24.6)	*13.1* ± 17.1 5.2 (2.4;19.9)	*17.2* ± 12.0 12.6 (10.4;25.9)
	Untreated sites	*10.5* ± 12.4 4.5 (1.6;15.8)	14.6* ± 16.5 9.3 (2.6;20.5)	*11.8* ± 13.0 10.0 (1.7;15.6)	*15.5* ± 14.2 16.1 (4.5;22.9)	*23.4* ± 18.2 31.2 (5.4;37.8)	*22.1* ± 12.9 26.7 (10.1;29.8)	*9.6* ± 8.1 9.4 (2.4;16.1)	*17.4* ± 12.3 20.7 (5.8; 27.8)
Soft tissue	Treated sites	*56.4* ± 16.2 59.3 (48.5;65.8)	*72.1* ± 19.6 69.0 (60.4;91.8)	*61.1* ± 15.8 65.6 (51.9;72.0)	*58.7* ± 7.2 58.3 (54.4;61.5)	*61.7* ± 11.5 58.8 (57.4;64.6)	*56.5* ± 7.5 56.6 (52.5;58.8)	*49.0* ± 16.7 51.9 (47.7;55.2)	*55.0* ± 8.1 55.2 (51.9;58.3)
	Untreated sites	*57.3* ± 15.6 55.4 (53.8;59.6)	*64.9* ± 22.5 68.5 (62.0;77.8)	*59.6* ± 16.6 59.3 (56.0;64.5)	*66.9* ± 11.8 69.1 (59.2;73.7)	*58.4* ± 9.3 61.0 (54.8;65.3)	*58.8* ± 3.3 59.2 (58.1;61.3)	*55.0* ± 8.4 *57.0* (52.0;59.8)	*59.0* ± 5.9 58.8 (57.7;60.4)

Data in percentages. Mean values in italics ± standard deviations. Medians (25th; 75th) percentiles. *p < 0.05

After 1 week (Table 1), the xenograft proportions in the antrostomy were 4.0 ± 7.5% in the treated and 43.5 ± 14.1% in the untreated sites (*p* = 0.012). After 8 weeks of healing (Table 2), the proportions decreased to 3.4 ± 4.9% and 11.8 ± 13.0% (*p* = 0.091), respectively.

After 1 week of healing (Table 1), in the close-to-window region, the proportions of xenograft were 21.3 ± 14.7% and 55.9 ± 19.0 (*p* = 0.012) in the treated and untreated sites, respectively. After 8 weeks of healing (Table 2), these values decreased to a similar percentage (15.5 ± 14.4% and to 15.5 ± 14.2%; *p* = 0.917, respectively).

### Grafted sinus regions

**Fig. 8:**
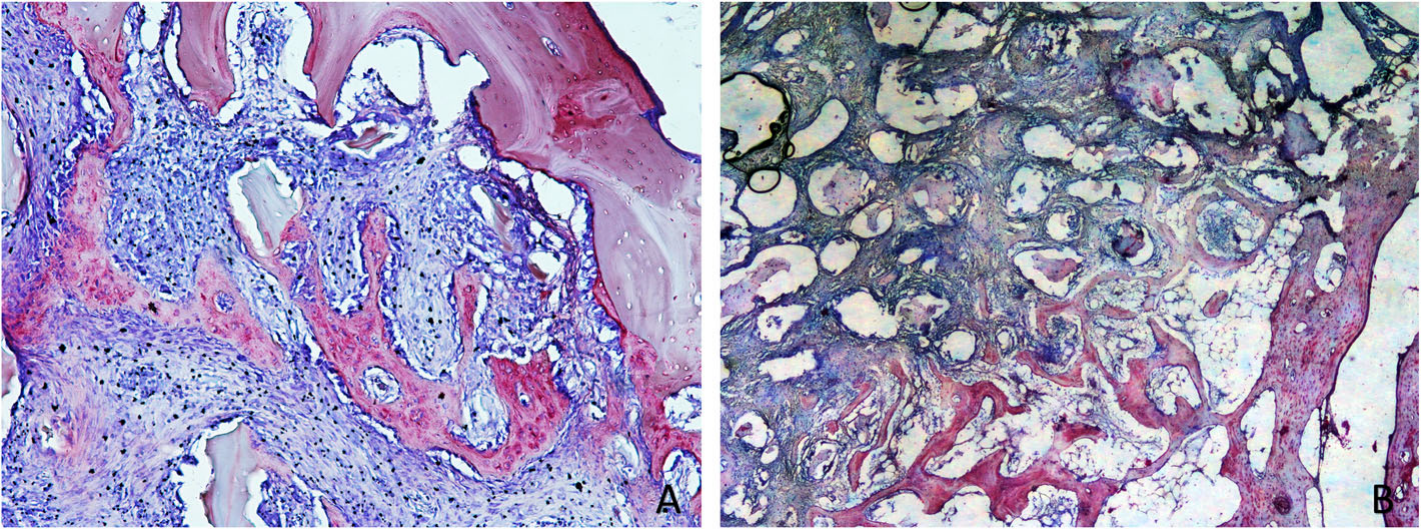
Photomicrographs of decalcified sections. **a** Untreated site. Woven bone formed from the sinus walls after 1 week of healing. **b** Treated site. After 8 weeks, woven bone was still found forming ridges towards residues of provisional matrix, showing that the healing was not completed yet. Scarlet-acid fuchsine and toluidine blue stain. **a** × 100 magnification. **b** × 20 magnification

After 1 week of healing (Table 1), in the grafted sinus the highest amount of newly formed bone was found in the bone walls region (Fig. 8a), while very little or no bone at all was found in the other regions. No differences were found between treated and untreated sites. After 8 weeks of healing (Table 2), new bone increased in both sites, reaching higher percentages in the treated compared to the untreated sites in all regions. The total proportion of bone was 27.9 ± 12.9% in the treated sites and 23.6 ± 15.2% in the untreated sites (*p* = 0.128). The highest percentages of bone were found in the bone walls regions, being 38.0 ± 15.0% and 35.4 ± 15.0% in the treated and untreated sites, respectively (p = 0.310). New bone was still found forming ridges towards the provisional matrix, showing that the healing was not completed yet in all regions and in all sinuses (Fig. 8b).

## Discussion

The present experiment aimed to study the influence on the healing after the placement of autogenous bone on the antrostomy and in the subjacent region after maxillary sinus elevation. After 8 weeks of healing, in the antrostomy region, a trend of higher bone formation in the treated compared to the untreated sites was observed. No statistically significant difference was found. In the subjacent region (close-to-window region), a higher content of new bone was found in the treated compared to the untreated region, being the difference statistically significant. A trend of higher proportions of new bone was observed in all the remaining regions within the elevated space in the treated compared to the untreated regions. However, none of the differences reached statistical significance.

The protection of the access window with membranes after sinus floor elevation using lateral access has been propagated as a procedure that yields higher implant success rate [3]. However, both clinical and experimental studies have shown that, despite the use of collagen membrane, complete closure of the antrostomies after sinus floor elevation might not be always achieved. In a clinical study [12], after 9 months from sinus floor elevation in which collagen membranes were used to cover the antrostomy, biopsies were harvested at the grafted sites from both the crestal region and the antrostomy. The histological analysis showed 40.1% of mineralized bone and 40.1% of marrow spaces in the biopsies retrieved from the alveolar crest while those from the antrostomy presented proportions of 26.0% and 23.4%, respectively. Moreover, only 3.9% of connective tissue was found in the crestal regions while in the antrostomy the respective proportion was 19.7%. This amount of connective tissue was mainly due to a few antrostomies that showed incomplete healing of the lateral window.

Experimental studies as well have reported incomplete healing of the antrostomies. In an experiment in sheep [19], at the test sites, perforation of the sinus mucosa was carried out and then protected with a collagen membrane. The elevated space was filled with biphasic calcium phosphate (60% HA/40% beta-TCP) on both test and control sites. The antrostomy was protected with a membrane made of polylactic acid and citric acid ester acetyl. After 12 weeks of healing, the bone was found formed from the margin of the antrostomy. However, none of the antrostomies was completely obliterated, presenting connective tissue occupying the central regions of the access window. In another similar experiment in sheep [20], a collagen membrane was placed only at the test sites subjacent to an intact sinus mucosa. The elevated space was filled with deproteinized bovine bone mineral (DBBM) and a collagen membrane was placed on the access window, both at the test and control sites. After 4 months of healing, the antrostomies of both sites presented high amounts of new bone (35–39%). Marrow spaces and residues of DBBM were found at proportions of 12–13% and 16–20%, respectively. However, connective tissue was found at high proportions in both sites (27–36%), interposed between the margins of the antrostomy.

In both studies presented above on sinus floor elevation in sheep, all the lateral windows were prepared using a piezoelectric device. In an experiment in rabbits [21], the antrostomies were done with either a sonic instrument or drills to evaluate differences in bone formation in the antrostomy. Elevated space is filled with a collagenated porcine bone similar that used in the present experiment, and both the antrostomies were covered with a collagen membrane. After 8 months of healing, no differences were found between the two sites. However, in a few specimens, the antrostomies presented and incomplete healing, with connective tissue in the central regions. In the antrostomies, new bone was found in a proportion of 28–29%, outcome similar to that observed in the present experiment in the untreated region after 8 weeks of healing (28.6%). However, in the treated antrostomy of the present study, new bone reached the proportion of 35.5%, showing that advantages might be obtained placing the autogenous bone in the antrostomy region, even though the difference was not statistically significant (*p* = 0.499). A statistically significant difference (*p* = 0.018) was instead found in the close-to-window regions in which new bone was found in proportions of 25.8% and 17.6% in the treated and untreated sites, respectively.

Aiming to improve the healing at both the elevated space and the antrostomy, precluding the ingrowth of connective tissue within the elevated region, the reposition of the bone window, removed after the osteotomy, has been proposed both in clinical [13, 14] and experimental studies [15, 16].

In a clinical study, 239 implants were installed simultaneously after 96 sinus floor elevation procedures performed in 84 patients [14]. The surgical procedures applied included the reposition of the bone window. After a follow-up of 1 to 6 years, a survival rate of 98.8% was obtained.

The osteogenic properties of the repositioned bone window have been shown in experiments in rabbits in which the maxillary sinus augmentation was performed bilaterally [15, 22]. In both studies, one antrostomy was covered using a collagen membrane while the other was closed repositioning the bone window. More new bone was found within the sinus covered with the repositioned bone window, formed in contact with its inner surface. In another study in rabbits [16], a similar experimental design was applied. Deproteinized bovine bone mineral was used as filler material in both sites, and the repositioned bone window was fixed with cyanoacrylate glue in the test group. A collagen membrane was applied in the control sites, and the healing was evaluated after 2, 4, and 8 weeks. No differences were found in the healing within the elevated space. In the antrostomy region of the test sites, the bone window was partly remodeled in the periphery and connected by bridges of newly formed bone to the inner regions of the grafted sinus. However, in the collagen membrane sites, the healing was incomplete in some specimens, presenting residual defects occupied by connective tissue. This agrees with the outcomes of the present study, that reported four antrostomies not closed and three closed, two of which presenting remaining defects. In the treated sites, two antrostomies were found not completely closed and five obliterated by bone, three of which with remaining defects.

The lower phylogenetic level of the animals compared to humans was the main limitation of the present study. An increased number of animals might allow reaching a statistical difference in favor of the treated sites also in the antrostomy region. Nevertheless, the outcomes obtained, allow to performing studies in humans that might demonstrate the advantages of applying autologous bone on the antrostomy.

Some features of autologous bone grafts may be relevant to the resorption process such as its form (block or particulate) [23], its microarchitecture (cortical or cancellous) [24], or the embryogenesis of the donor sites (intramembranous or endochondral ossification) [25].

The present study harvested autologous bone from the rabbit tibia, i.e., endochondral ossification. In humans, this type of ossification has demonstrated less dimensional stability than intraoral grafts that present intramembranous ossification [24, 25]. For this reason, and because of the less patient morbidity and risk of complications, intraoral autogenous bone harvesting is routinely used [26, 27]. Intraoral particulated bone (intramembranous origin) can be obtained from the cortical bone of the lateral window [28] or from neighbor intraoral areas (e.g., from the ramus, or the chin, or retromolar area). The difference in the origin of the autograft should be taken into consideration when interpreting the results of the present preclinical study.

It can be concluded that the placement of autogenous bone in the antrostomy and the subjacent region after maxillary sinus elevation, provides slightly better new bone formation compared with sites only grafted with xenograft. Though, only the subjacent close-to window region showed a statistical significance within treated sites at 8 weeks of healing. Despite the limitations of the present study and due to its preclinical nature, results should be extrapolated to humans with caution.

## Data Availability

The datasets used or analyzed during the current study are available from the corresponding author on reasonable request.

## Abbreviations

ARRIVE: Animal Research Reporting In Vivo Experiments
CBCT: Cone beam computed tomography
DBBM: Deproteinized bovine bone mineral
TCP: Tricalcium phosphate
